# ModuleMiner - improved computational detection of *cis*-regulatory modules: are there different modes of gene regulation in embryonic development and adult tissues?

**DOI:** 10.1186/gb-2008-9-4-r66

**Published:** 2008-04-07

**Authors:** Peter Van Loo, Stein Aerts, Bernard Thienpont, Bart De Moor, Yves Moreau, Peter Marynen

**Affiliations:** 1 Department of Molecular and Developmental Genetics, VIB, Herestraat 49, B-3000 Leuven, Belgium; 2 Department of Human Genetics, University of Leuven, Herestraat 49, B-3000 Leuven, Belgium; 3 Bioinformatics group, Department of Electrical Engineering (ESAT-SCD), University of Leuven, Kasteelpark Arenberg, B-3001 Heverlee, Belgium

## Abstract

We present ModuleMiner, a novel algorithm for computationally detecting *cis*-regulatory modules (CRMs) in a set of co-expressed genes. ModuleMiner outperforms other methods for CRM detection on benchmark data, and successfully detects CRMs in tissue-specific microarray clusters and in embryonic development gene sets. Interestingly, CRM predictions for differentiated tissues exhibit strong enrichment close to the transcription start site, whereas CRM predictions for embryonic development gene sets are depleted in this region.

## Abstract 2

ModuleMiner detects *cis*-regulatory modules in a set of co-expressed genes in tissue-specific microarray clusters and in embryonic development datasets.

## Background

The identification and functional annotation of transcriptional regulatory sequences in the human genome is lagging far behind the rapidly increasing knowledge of protein-encoding genes. These transcriptional regulatory sequences are often build up in a modular manner and exert their function in *cis *through the concerted binding of multiple transcription factors (and co-factors), resulting in the formation of protein complexes that interact with RNA polymerase II [1,2]. These sequences are called *cis*-regulatory modules (CRMs). In theory, these CRMs can be detected by the presence of multiple transcription factor binding sites (TFBSs). In practice, however, reliable detection of functional TFBSs is difficult and results in many false positives, partly because these binding sites are too short and too degenerate [3]. Hence, the computational detection of functional regulatory sequences in the human genome remains a formidable challenge.

Multiple methods have been developed that aim to detect regulatory sequences computationally [4-8]. Promising and validated results have been delivered mostly in model organisms with relatively compact genomes (for example, *Drosophila melanogaster*) [9-11]. In the larger human genome, deep sequence conservation (for instance, up to zebrafish) or extreme sequence conservation (for example, perfect conservation in mouse over 200 base pairs), irrespective of TFBS detection, remains the method of choice for approaches validating regulatory sequences *in vitro *or *in vivo *[12-14]. Although these conservation approaches are quite successful in predicting which regions have a regulatory function, they provide no information regarding what expression pattern these regions produce and by which transcription factors they are targeted.

When several similar CRMs have been characterized, and the regulatory factors and binding sites have been elucidated, one can use this knowledge to find new examples of similar CRMs that direct the transcription of other genes that are involved in the same process. A number of computational methods have been described that apply this approach [15-17]. These methods have been highly successful [10,11,18], but in practice - apart from in *Drosophila *embryonic development - the lack of available data often precludes the application of these approaches.

When this knowledge is not available, the detection of tissue-specific or process-specific CRMs can be tackled by looking for recurring combinations of TFBSs in putative regulatory regions of a set of co-expressed genes. A few methods applying this approach have been developed [19-22]. However, partly because this is a more complex problem, these methods have only been applied on a limited scale and few successful predictions have been reported. To our knowledge, our ModuleSearcher method [20] is the only one to have yielded results that have undergone experimental validation [23].

Here, we develop ModuleMiner, a novel algorithm designed to detect similar CRMs in a set of co-expressed genes, focused on the human genome. ModuleMiner does not require prior knowledge of regulating transcription factors or annotated binding sites, but uses only a library of position weight matrices (PWMs). Contrary to existing algorithms, which require *a priori *knowledge of CRM properties (such as the length of the CRMs or the number of binding sites) as input parameters, ModuleMiner requires no parameters. In addition, ModuleMiner differs from existing similar approaches in that it implements a whole-genome optimization strategy to look specifically for signals that discriminate the given co-expressed genes from all other genes in the genome. By leave-one-out cross-validation on benchmark data, we show that ModuleMiner outperforms other methods that computationally detect CRMs. Finally, we demonstrate that ModuleMiner can successfully detect similar CRMs in microarray clusters with a tissue-specific expression profile, as well as in custom-build gene sets related to specific embryonic developmental processes. In total, ModuleMiner predicted 257 CRMs near to the genes studied, as well as an additional 1,400 CRM predictions resulting from full genome scans for new target genes. We further analyze these CRM predictions to elucidate differences between CRMs directing transcription in differentiated tissues and CRMs directing transcription during embryonic development.

## Results

### ModuleMiner: detection of similar CRMs in a set of co-expressed genes

We developed ModuleMiner, a novel algorithm to detect similar CRMs in a set of co-expressed genes. ModuleMiner models similar CRMs as a combination of motifs (represented by PWMs) in the same way as in the report by Aerts and coworkers [20]. These models are called 'transcriptional regulatory models' (TRMs) [24]. We postulate that a good TRM can retrieve targets in the genome. Therefore, we express the fitness of a TRM in terms of its target gene recovery and we select the TRM that has maximum specificity for the given set of co-expressed genes, using a whole-genome optimization strategy. To determine the fitness of a TRM, each gene's search space is first scored with the TRM, where we define a gene's search space as the collection of all conserved noncoding sequences within 10 kilobases (kb) 5' of the transcription start site (TSS; see Materials and methods, below). These scores are then used to rank all genes in the genome. Finally, the ranks of the given co-expressed genes are determined, and the probability of observing this collection of ranks by chance is calculated using order statistics (see Materials and methods, below). If a large part of the co-expressed genes are ranked high, then the order statistic is highly significant, and hence the TRM is considered to have a high fitness for modeling similar CRMs that regulate these genes. ModuleMiner searches the TRM with the most significant order statistic (the best fitness) using a genetic algorithm (detailed in Materials and methods, below).

We introduce ModuleMiner and its rigorous validation procedure using an example case study. We constructed a high-quality set of 12 smooth muscle marker genes [25], and performed leave-one-out cross-validation (LOOCV). In each validation run, one gene was left out and ModuleMiner constructed a TRM using the remaining 11 genes. This TRM was then used to rank all genes in the genome and the position of the left-out gene was determined. The set of 12 ranks obtained in this way was used to calculate sensitivity/specificity pairs, which were subsequently plotted on a receiver operating characteristic (ROC) curve. We used the area under the ROC curve (AUC) as a measure of ModuleMiner's performance on this set of co-expressed genes.

**Figure 1:**
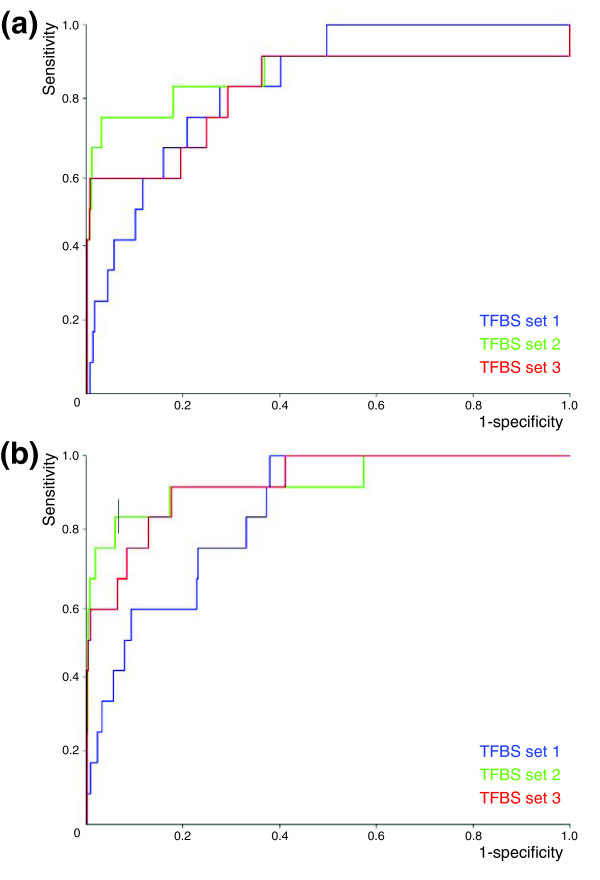
Performance of ModuleMiner. Illustrated is the performance of ModuleMiner on a set of smooth muscle marker genes, using the three different sets of candidate transcription factor binding sites (TFBSs). Receiver operating characteristic curves are shown, representing results for leave-one-out cross-validations on the set of smooth muscle markers, **(a) **using singular transcriptional regulatory models and **(b) **using transcriptional regulatory global models.

We repeated the LOOCV for three sets of candidate TFBSs (Table 1). The first set includes predicted binding sites in human-mouse conserved noncoding sequences (CNSs), obtained by aligning 10 kb 5' of all human-mouse orthologs and selecting regions of at least 75% identity over a minimum of 100 base pairs. The second set includes a refined series of binding sites from the first set; specifically, it retains only the PWMs for which an instance is predicted in both human and mouse CNSs (we follow the nomenclature presented by Berman and coworkers [10] and call these sites *'preserved' *sites). Finally, the third set is refined further from the second set; specifically, the CNSs are obtained by aligning 10 kb 5' of all human genes to 110 kb 5' + 100 kb 3' of the TSS of their mouse orthologs (and hence correcting for possible differences in TSS annotation). The resulting ROC curves are shown in Figure 1a. In all three cases, the AUC values are significantly above 50% (the theoretical value obtained if the left-out genes were ranked randomly), indicating that the TRMs obtained are sensitive and specific in predicting CRMs near to the left-out genes.

**Table 1:** Genome-wide databases of candidate transcription factor binding sites

Number	Database properties	Number of genes	Number of regions	Number of binding sites
1	Human-mouse conserved regions, 10 kilobases 5' of TSS	8,759	22,582	1,858,800
2	(1) + limited to binding sites occurring both in the human and mouse CNS	8,759	22,582	878,338
3	(2) + correct for possible mouse TSS differences (add 100 kilobases of mouse sequence 5' and 3')	11,653	35,021	1,316,927

CNS, conserved noncoding sequence; TSS, transcription start site.

We observed that similar TRMs have similar fitness and similar order statistic. The TRM that is selected by ModuleMiner (the one that has the lowest order statistic) is surrounded by similar TRMs with order statistics that are only slightly larger. The selection of one TRM out of these similar TRMs is inherently arbitrary and depends only marginally on the true regulatory signals. To make ModuleMiner more robust to this 'noise', we cluster the top-scoring TRMs and select the most prominent cluster instead of the single optimal TRM. We call this cluster of TRMs a 'transcriptional regulatory global model' (TRGM). The results of a LOOCV when using these TRGMs (Figure 1b) show that this indeed has a positive effect on ModuleMiner's performance: the AUCs increased by 6% on average. Furthermore, these TRGMs provide additional information compared with singular TRMs, because they allow an estimate of the relative importance of each PWM involved, as discussed below.

When comparing the performance of ModuleMiner (using TRGMs) on the three sets of candidate binding sites, a large difference between selecting all detected binding sites (set 1: AUC value 84.6%) and restricting to preserved sites only (set 2: AUC value 92.8%) is apparent. Correcting for TSS differences in human and mouse (set 3: AUC value 92.5%) did not increase this performance further. Thus, for this high-quality set of co-expressed genes, the preservation of binding sites is highly beneficial for efficient detection of CRMs. This strongly suggests that for this gene set the trans-acting factors are conserved between human and mouse.

We next applied the ModuleMiner algorithm to the full set of 12 smooth muscle marker genes, using the site preservation measure (set 2). The resulting TRGM identifies SRF, SMAD4, SP1, and ATF3 as the main transcription factors involved in the co-regulation of these genes (detailed ModuleMiner output is reported on our website [26]). Importantly, ModuleMiner implicates SRF as the most important smooth muscle regulator, and suggests that smooth muscle specific regulation often entails two or more SRF binding sites, which is in agreement with the literature [27].

To verify the added value of the resulting combination of PWMs over SRF alone, we manually generated a TRGM containing only PWMs for SRF, and compared the performance of this model with that of ModuleMiner. When we applied this 'SRF only' TRGM to rank the genome, we obtained an AUC of 79.9%, which is significantly smaller than the 92.8% AUC of ModuleMiner (obtained in an LOOCV setting).

### Sensitivity to noise

To assess the performance of ModuleMiner as a function of the composition of the input set of co-expressed genes, we performed LOOCV on input sets that contain a varying percentage of genuinely co-regulated genes ('true positives'). As true positive genes, we selected the set of ten smooth muscle markers that share similar CRMs that can be identified by ModuleMiner (these ten genes all are ranked within the top 7% of the genome by a LOOCV, as shown in Figure 1b). We approximated negative genes (genes that do not contain the smooth muscle CRM) by random genes.

**Figure 2:**
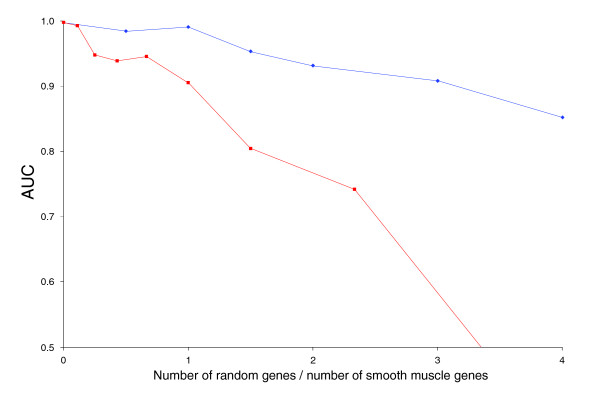
Sensitivity of ModuleMiner's performance to the quality of the input genes. The ratio of true positive genes (containing the smooth muscle *cis*-regulatory module [CRM]) to negative genes (approximated by random genes) was varied. Each time, a leave-one-out cross-validation was performed, a receiver operating characteristic (ROC) curve was constructed, and the area under the ROC curve (AUC) was calculated. These AUCs were plotted as a function of the ratio negative genes/positive genes. Because an AUC of 50% signifies random ordering of the left-out genes (and hence indicates that no CRMs can be detected), this value was taken as the origin on the y-axis. Blue: the number of positive genes was kept constant at ten, and the number of negative genes was varied. Red: the total number of genes was kept constant at ten, and the ratio negative genes/positive genes was varied.

In a first analysis, we kept the number of true positive genes constant at ten, and we added a varying number of negative genes. The decrease in performance as a function of an increasing number of negative genes was surprisingly small (Figure 2). Even when only 10 out of 50 genes contained the smooth muscle CRM, ModuleMiner was able to pick up this signal (the AUC was 85.2%, and SRF and SP1 were still identified as key factors).

In a second analysis, we kept the total number of genes constant at ten, and we varied the percentage of negative genes. We now observed a steep decrease in ModuleMiner performance as a function of an increasing percentage of negative genes (Figure 2).

We conclude from these experiments that ModuleMiner requires a critical mass of true positive genes for successful detection of similar CRMs. However, when this critical mass is present, ModuleMiner is highly robust to false-positive genes.

### Comparison with other CRM detection algorithms

We next compared ModuleMiner with other *in silico *approaches for CRM detection on benchmark data. From PAZAR [28], we selected all 'boutiques' containing annotated regulatory regions directing expression in a particular system: M02, muscle; M03, liver; M08, ORegAnno Stat1; and M09, ORegAnno Erythroid. As a fifth benchmark set, we used the 12 smooth muscle genes described above. On each of these five sets, we compared the performance of ModuleMiner with that of four state-of-the-art publicly available algorithms designed to detect similar CRMs in co-expressed genes: ModuleSearcher [29], CREME [19], CisModule [22], and EMCMODULE [30]. We also included the Clover algorithm [31], which looks for individual over-represented TFBSs in putative regulatory sequences of a set of co-expressed genes. We note that our analysis does not focus specifically on the known enhancers, but in contrast we consider all CNSs in the entire 10 kb 5' of the TSS (which may or may not contain the known enhancer, as well as other sequences). This effectively mimics a real-life situation, where the exact location of the regulatory sequences is not known *a priori*.

The CREME algorithm was unable to identify similar CRMs in any of the five benchmark sets, most likely in part because of its focus on larger sets of more loosely co-expressed genes [19]. Using the remaining algorithms, we performed LOOCV on each of the five benchmark sets. For this LOOCV, we used each algorithm to train a TRM or TRGM using gene sets in which one gene is left out (see Materials and methods, below, for details). Hence, as training data, we used all CNSs in the 10 kb 5' of the TSS of the benchmark set, except for the left-out gene. For CisModule and EMCMODULE, the inputs were the sequences of the CNSs; for Clover, the inputs where the sequences of the CNSs as well as all TRANSFAC and JASPAR vertebrate PWMs; for ModuleSearcher, the inputs were the predicted binding sites within those CNSs, using all TRANSFAC and JASPAR vertebrate PWMs. The combination of PWMs that each algorithm provided as output was used to build a TRM or TRGM. We subsequently used the ModuleScanner algorithm to rank all genes in the genome based on the predicted TRM/TRGM, and we used the results to construct ROC curves. We used the site preservation measure (candidate TFBS set 2) for the ModuleMiner runs (because this was the set in which we obtained the best results for the smooth muscle genes). Because the other algorithms do not use site preservation in the discovery step, we used candidate TFBS set 1 (without preservation) also in their genome ranking step. We also constructed random ROC curves based on genome ranking using random TRMs (see Materials and methods, below, for details).

**Figure 3:**
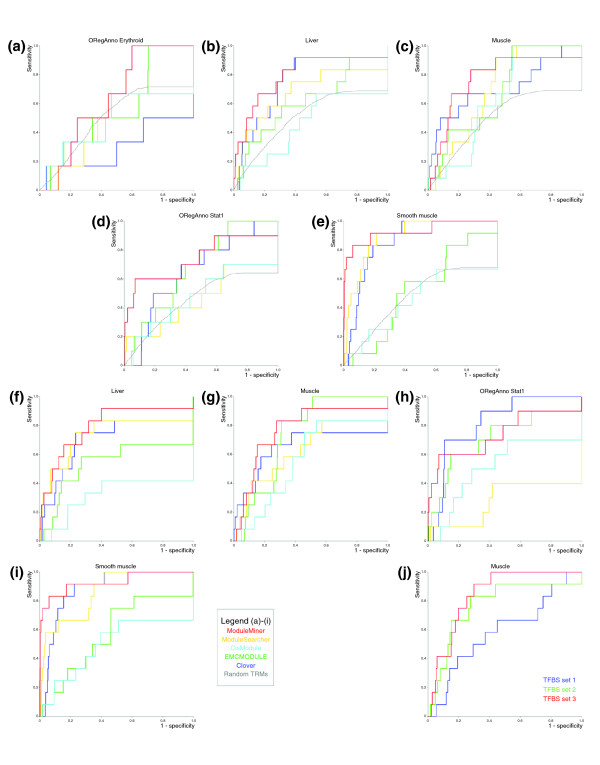
Comparison with other CRM detection algorithms. **(a-e) **Receiver operating characteristic (ROC) curves for the leave-one-out cross-validation using ModuleMiner, ModuleSearcher, CisModule, EMCMODULE, Clover, and random transcriptional regulatory models for each of the five benchmark sets: ORegAnno Erythroid (panel a), liver (panel b), muscle (panel c), ORegAnno Stat1 (panel d) and smooth muscle (panel e). **(f-i) **ROC curves when using transcription factor binding site (TFBS) preservation (TFBS set 2) in the genome ranking step for all algorithms, on the four benchmark sets that performed above random: liver (panel f), muscle (panel g), ORegAnno Stat1 (panel h), and smooth muscle (panel i). **(j) **ModuleMiner performance for the three TFBS sets on the muscle benchmark data. CRM, *cis*-regulatory module.

On the OregAnno Erythroid benchmark set neither ModuleMiner nor any of the other algorithms appear to perform better than random (Figure 3a). Because this is the smallest set, containing only six genes with human-mouse CNSs, this is consistent with the results we obtained in the previous section, in which we concluded that a critical number of co-regulated genes is required for CRM detection. In contrast, on each of the four other benchmark sets, ModuleMiner performs better than random TRMs, as do some of the other algorithms (Figure 3b-e). Comparing the performance of all CRM detection algorithms, ModuleMiner appears to exhibit the best performance in all four cases. Interestingly, only ModuleMiner can compete with 'simple' TFBS over-representation in this setup, emulating a real-life situation in which the regulatory sequences are not known. Indeed, only ModuleMiner outperforms Clover on four of the five benchmark sets. On the fifth benchmark set (muscle), Clover and ModuleMiner seem to be closely matched, with the Clover method showing a steeper start of the ROC curve.

The performance of the other CRM detection algorithms can be improved by using site preservation (TFBS set 2) in the genome ranking step (Figure 3f-i), although ModuleMiner outperforms all other CRM detection algorithms here also, which suggests that the TRMs predicted by ModuleMiner are more informative or more specific than those suggested by other methods. Candidate TFBS set 2 was not in all cases the optimal choice for ModuleMiner; on the muscle benchmark set, candidate TFBS set 3 performed better (Figure 3j).

We noticed that the CRM predictions ModuleMiner made on the muscle, liver, and ORegAnno Stat1 sets correspond well with the known regulatory elements. The TRGMs ModuleMiner contructed contain PWMs for SRF, MEF2, Myf and MyoD (muscle), HNF1, HNF3, HNF4 and CEBP (liver), and STAT (ORegAnno Stat1), even though we used all CNSs in the 10 kb upstream region. In addition, the CRM predictions mostly overlap the true enhancer, when the real regulatory sequence was in our CNS collection. Indeed, for the muscle set, in 9 of the 11 cases in which the known enhancer was in our CNS set, ModuleMiner was ably to identify this region. For the liver set, ModuleMiner identified seven out of eight regulatory elements (data not shown).

### Detection of CRMs in microarray clusters

Realizing that clustering of microarray data provides a rich source of large co-expressed gene sets, in which robustness to genes that are not co-regulated ('false positive genes') is critical, our sensitivity to noise analysis above encouraged us to apply ModuleMiner to microarray clusters on a larger scale. The GNF SymAtlas [32] contains expression profiles of 140 human and mouse tissues. Nelander and coworkers [33] obtained gene clusters by hierarchically clustering this dataset, followed by a Pearson's correlation coefficient cut-off. From this clustering, we selected all clusters with at least 25 genes in our dataset (genes with at least one CNS within 10 kb 5' of the TSS). This results in ten clusters with sizes ranging from 26 to 214 genes. Large clusters were randomly divided in a training set of 50 genes, and a test set containing the remaining genes.

**Figure 4:**
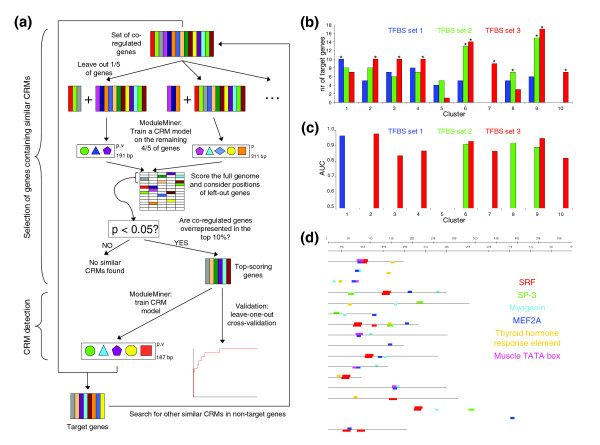
Application of ModuleMiner to microarray clusters. **(a) **The two-step procedure used to detect similar *cis*-regulatory modules (CRMs) in a subset of genes within a given microarray cluster. In the first step, a fivefold cross-validation is performed, and the number of left-out genes considered as target genes is counted. If this number is significantly more than expected under a random distribution of the ranks, then these genes are transferred to the second step. In this second step, ModuleMiner is used to model the similar CRMs regulating the genes in this focused subcluster. **(b) **Results of the first step of the procedure in panel (a) for the ten microarray clusters and the three different sets of candidate transcription factor binding sites (TFBSs). Significantly higher numbers of target genes among the left-out genes than randomly expected are depicted by an asterisk. Clusters 7 and 10 only contained sufficient genes (≥ 25) in TFBS set 3 and therefore are omitted for the other two sets. **(c) **Leave-one-out cross-validation results on the subclusters with a significant enrichment of target genes from panel (b). Each left-out gene was ranked using the transcriptional regulatory global model (TRGM) obtained on the remaining genes. Next, sensitivity/specificity pairs where calculated for different detection thresholds, and these were used to construct receiver operating characteristic (ROC) curves. The areas under these ROC curves (AUCs) were calculated and are depicted here. The colors are as in panel (b). **(d) **Presented is an example of a set of similar CRMs identified by ModuleMiner. These results were obtained on the cardiac muscle genes by the procedure depicted in panel (a). Each horizontal line represents a human-mouse conserved noncoding sequence (CNS) upstream of a gene within the cluster. The different colored boxes represent binding sites of different transcription factors. Detailed results, including descriptions of the genes shown, and the exact positions of the CNSs are available on our website [26].

Because it was our goal here to identify similar CRMs within a subset of the genes in each microarray cluster, we used a two-step procedure. First we detected which subset of genes potentially share CRMs, and next we detected the actual CRMs in their upstream regions (Figure 4a). The first step consisted of a fivefold cross-validation, where in each validation run we used ModuleMiner to train a TRGM on four-fifths of the genes in a cluster, and next we determined which of the other one-fifth of left-out genes were targets of the TRGM. If the total number of true target genes among left-out genes was not significantly higher than random, then we concluded that ModuleMiner is unable to detect similar CRMs within this cluster. If on the other hand there was a significant enrichment of these true target genes, then we concluded that ModuleMiner can detect similar CRMs, and we used these high scoring genes in the second step. In this second step, ModuleMiner was applied to this focused subcluster, identifying similar CRMs that regulate these genes. As an extra validation, LOOCV was used to confirm the presence of similar CRMs, as done previously on the smooth muscle and other benchmark sets.

Application of this procedure to the microarray clusters described above resulted in successful CRM detection in nine out of the ten clusters (Table 2 and Figure 4b). In each case, this success was confirmed by a LOOCV on the selected subcluster (all AUCs were significantly above 50%, with an average AUC of 90.3%; Figure 4c). For the TRGMs obtained for clusters containing more than 50 genes, the number of targets in the independent test set was determined. This was significantly higher than random in three of the five cases (Table 2). In total, we predicted 209 CRMs. These ModuleMiner predictions can be viewed in detail on our website [26].

**Table 2:** Summary of ModuleMiner's results for the ten microarray clusters

Cluster	Annotation	TFBS set	Number of target genes after cross-validation (*P*)	AUC on target genes	Number of target genes in independent test set (*P*)	Total number of CRMs
1	Protein synthesis	1	10/50 (0.025)	0.96	14/123 (0.35)	30
2	Oocyte/fertilized egg	3	10/50 (0.025)	0.98	30/164 (8.6 × 10^-4^)	43
3	Neural tissues	3	10/50 (0.025)	0.84	15/122 (0.24)	29
4	Lymphocytes	3	10/50 (0.025)	0.87	23/85 (7.0 × 10^-6^)	36
5	Testis/spermatogenesis	-	-	-	-	-
6	Liver	3	14/50 (2.9 × 10^-4^)	0.93	7/29 (0.022)	23
7	Mitochondrion	3	9/31 (0.0026)	0.87	-	12
8	Extracellular matrix	2	7/32 (0.036)	0.92	-	10
9	Cardiac muscle	3	17/32 (6.6 × 10^-10^)	0.95	-	16
10	Energy metabolism	3	7/26 (0.012)	0.82	-	10

Transcription factor binding site (TFBS) sets: set 1 includes human-mouse conserved noncoding sequences (CNSs) 10 kilobases 5' of the transcription start site (TSS); set 2 includes set 1 + binding site preservation; and set 3 includes set 2 + correction for TSS differences. For clusters in which multiple TFBS sets resulted in successful *cis*-regulatory module (CRM) detection, only the result showing the best cross-validation performance is shown. Genes (in the cluster) that by cross-validation were ranked within the top 10% of the genome where considered target genes of the transcriptional regulatory global model (TRGM). The total number of CRMs constitutes all successful CRM predictions near to genes in the cluster. CRM predictions were considered successful if the TRGM score was sufficient to rank the target gene within the top 10% of the genome. In some cases, multiple CRMs are found that control the same target gene.

### Detection of CRMs in embryonic development gene sets

In the previous section we detected CRMs in microarray clusters expressed in different adult tissues. Next, we aimed to predict CRMs involved in embryonic development processes.

We constructed five gene sets involved in specific embryonic development processes, based on the literature (Table 3). Contrary to the previous section, in which we aimed to detect similar CRMs in a subset of the genes in the microarray clusters (using a two-step approach), here we can assume that the embryonic development gene set is more focused, and hence we can directly apply ModuleMiner to these sets (as in our high-quality smooth muscle gene set). We performed LOOCV, confirming that ModuleMiner was able to successfully detect similar CRMs in all five gene sets (Table 3).

**Table 3:** Summary of ModuleMiner's results for the five embryonic development gene sets

Embryonic development process	TFBS set	Number of target genes after LOOCV (*P*)	AUC
Primary heart field [50]	1	6/7 (6.4 × 10^-6^)	0.92
Secondary heart field [50]	1	6/9 (6.4 × 10^-5^)	0.79
Neural crest cells [51]	2	6/10 (1.5 × 10^-4^)	0.86
Eye development [52]	1	10/15 (1.9 × 10^-7^)	0.79
Limb development [53]	1	10/24 (5.2 × 10^-5^)	0.77

A key review or book used as a basis for construction of the development gene set is given in the first column. The genes in each set as well as the detailed results can be viewed at our website [26]. Transcription factor binding site (TFBS) sets: set 1 includes the human-mouse conserved noncoding sequences (CNSs) 10 kilobases 5' of the transcription start site (TSS); set 2 includes set 1 + binding site preservation; and set 3 includes set 2 + correction for TSS differences. For clusters where multiple TFBS sets resulted in successful *cis*-regulatory module (CRM) detection, only the result showing the best cross-validation performance is shown. Genes (in the cluster) that by cross-validation where ranked within the top 10% of the genome where considered target genes of the transcriptional regulatory global model. LOOCV, leave-one-out cross-validation.

### Characterization of the CRMs

The TRGMs that were predicted by ModuleMiner in each of the ten microarray clusters and each of the five embryonic development gene sets are summarized in Tables 4 and 5. Apart from this TRGM, ModuleMiner also provides additional information characterizing the CRMs. We shall discuss here the results we obtained in cluster 9, which contains genes related to cardiac muscle function.

**Table 4:** Transcriptional regulatory global models constructed for the ten microarray clusters

Cluster	Key transcription factors and binding sites in TRGM (weight)
Protein synthesis	NF-Y (1.59), DEC (1.13), HIC1 (1.09), general initiator sequence (0.47), CCAAT box (0.44), TCF-4 (0.32)
Oocyte/fertilized egg	T3R (1.00), NF-Y (1.00), ETS/PEA3 (0.99), MAZ (0.92), AP2α (0.78), SP1 (0.30)
Neural tissues	UF1-H3β (1.13), CRE-BP/CJUN/ATF-1 (1.00), AP-2 (0.87), ETF (0.55), AP-1/NF-E2 (0.33)
Lymphocytes	STAT6 (1.00), PU.1 (0.99), ETS (0.96), STAT5/STAT (0.95), SP1 (0.89)
Testis/spermatogenesis	-
Liver	TCF1/HNF-1 (1.00), NF-1 (1.00), C/EBP (0.99), HNF-4/COUP (0.99), PPAR/HNF-4/COUP/RAR (0.66), MYC-MAX (0.58), PPAR (0.33)
Mitochondrion	c-ETS (1.35), VDR (1.00), GATA-1/GATA-2 (1.00), ZID (0.82), AR (0.43), ROAZ (0.34)
Extracellular matrix	AP-1/NF-E2/BACH1 (2.00), FOXD1 (1.00), BLIMP1 (1.00), SRF (0.70), MEF-2/RSRFC4 (0.51), STAT5/STAT6 (0.35)
Cardiac muscle	SP-3 (1.00), myogenin (1.00), MEF2A (1.00), SRF (1.00), tyroid hormone receptor/RAR/RXR (0.91), muscle TATA box (0.48)
Energy metabolism	CREB/ATF/HLF (1.01), WHN (1.00), SPIB (0.71), PPARγ/RXRα (0.65), general initiator sequence (0.51), RFX (0.31)

TRGM, transcriptional regulatory global model.

**Table 5:** Transcriptional regulatory global models constructed for the five embryonic development sets

Development process	Key transcription factors and binding sites in TRGM (weight)
Primary heart field	D type LTRs (1.12), HAND1/TCF3 (1.01), STAT3 (0.92), STAT5A (0.89), GATA1/GATA2 (0.63), ELK1 (0.32)
Secondary heart field	HNF3α (1.56), STAT5A/STAT5B (1.00), GATA2 (0.56), NFAT (0.56), GATA/GATA3 (0.48), WHN (0.35)
Neural crest cells	FREAC-7 (1.00), Poly A (1.00), TBX5 (1.00), HSF (0.89), FREAC-2 (0.30)
Eye development	RREB1 (1.00), IRF (0.96), POU3F2 (0.92), ZF5 (0.80), GATA/GATA1 (0.46), LMO2 (0.39), NKX6-1 (0.32)
Limb development	TEF (1.00), PLZF (1.00), PAX4 (0.96), EGR (0.87), AP-2 (0.65), PBX (0.63), Ikaros 1 (0.37)

TRGM, transcriptional regulatory global model.

First, ModuleMiner characterizes the given input genes, retrieving descriptions and commonly used identifiers (for example, HGNC) from the Ensembl database. In addition, the Gene Ontology (GO) terms annotated to the input genes are retrieved, and the over-represented GO terms are reported. For the cardiac muscle subcluster 'muscle contraction' (GO:0006936), 'muscle development' (GO:0007517), 'organogenesis' (GO:0009887), 'contractile fiber' (GO:0043292), and 'regulation of heart contraction rate' (GO:0008016) were among the over-represented GO terms.

Next, ModuleMiner determines the weight of each PWM in the TRGM (see Materials and methods, below). By grouping similar PWMs, the weight of each trans-factor involved is determined. The cardiac muscle TRGM contains PWMs for SRF, MEF2A, myogenin, SP3, a thyroid hormone response element (all with weights of approximately 1), and a muscle TATA box (with weight approximately 0.5). ModuleMiner also displays the CRMs that it identifies on the input genes. Figure 4d shows this for the heart muscle genes.

Because our approach uses only human and mouse sequences to model CRMs, sequenced genomes of other species can be used as validation data. ModuleMiner employs the rat and dog genomes for this purpose, by checking for CRMs that fit the obtained TRGM in rat-dog CNSs. For the cardiac muscle genes, 11 orthologs were present in our rat-dog TFBS database, seven of which were ranked within the top 10% of the genome (*P *= 2.28 × 10^-5^).

Finally, ModuleMiner selects putative new target genes of the TRGM from the complete genome. We aim to minimize noise in these target gene predictions by using network level conservation [34], particularly through phylogenetic fusion of target gene rankings. To this end, first all genes in the human-mouse TFBS database (excluding the input genes) and all (noninput) genes in the dog-rat TFBS database are ranked separately. ModuleMiner then fuses these two rankings into one global ranking using order statistics (similar to the approach used by Aerts and coworkers [23,35]). Among the 100 top ranking new target genes of the cardiac muscle TRGM were MYL3 ('cardiac myosin light chain 1'), MYOD1 ('myoblast determination protein 1'), TNNI1 ('troponin I'), and MYH3 ('myosin heavy chain, embryonic skeletal muscle').

The results we obtained on all sets of co-expressed genes discussed in this work can be viewed on our website [26].

### Where are the CRM predictions located?

ModuleMiner successfully detected nine sets of similar CRMs in the ten microarray clusters and five sets of similar CRMs in the five embryonic development gene sets. In total, 257 CRMs were predicted. In addition to this, ModuleMiner predicted 100 new target genes of each TRGM. We next used this compendium of 1,657 CRMs to examine their positions relative to the TSSs of the genes that they regulate.

**Figure 5:**
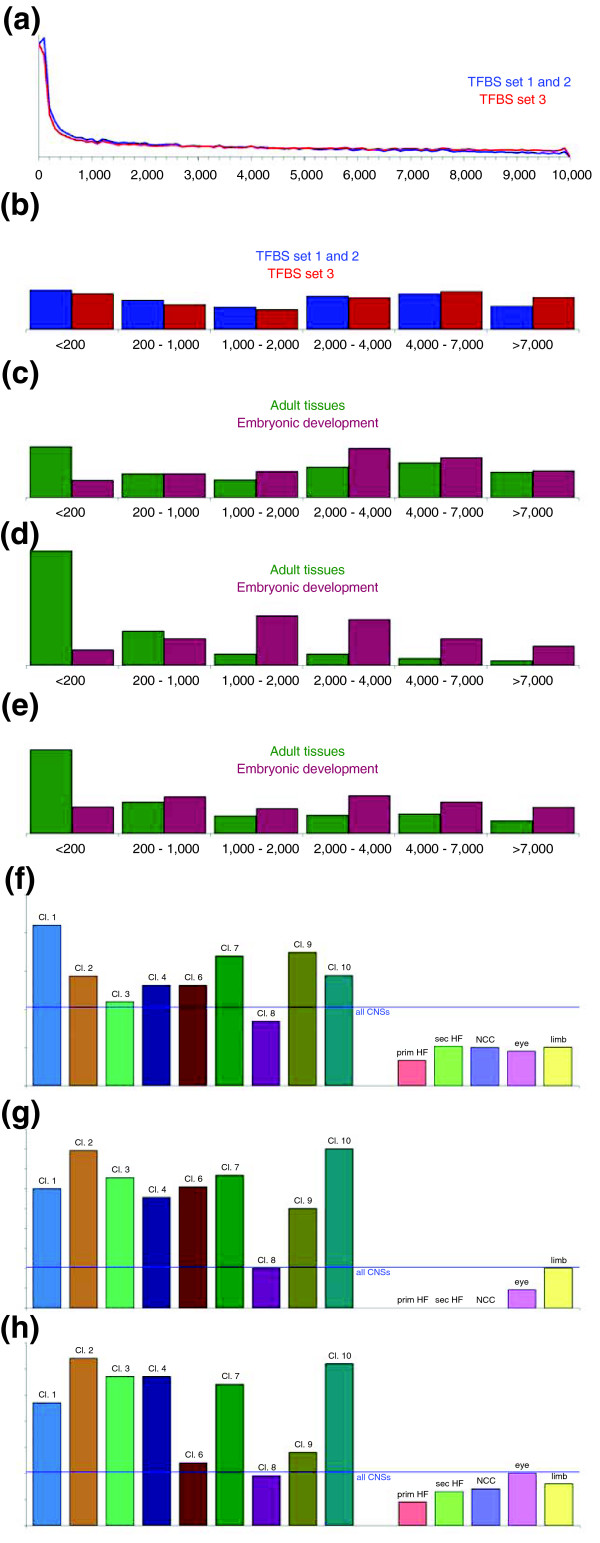
Distribution of distance to transcription start site for CNSs and predicted CRMs. **(a) **All human-mouse conserved noncoding sequences (CNSs) in transcription factor binding site (TFBS) sets 1 and 2 (both are based on the same set of CNSs) and in TFBS set 3. **(b) **The distribution from panel (a), when divided into six unequal bins. **(c) **Distribution of all CNSs upstream of genes within the microarray clusters (of genes expressed in different adult tissues) and the embryonic development gene sets, where CRMs could successfully be detected (Tables 2 and 3), divided into the same six bins as under panel (b). **(d) **Distribution of the distance to transcription start for the CRMs that ModuleMiner identified near to the genes from panel (c). **(e) **Distribution of distance to transcription start for the CRMs that ModuleMiner identified in a whole genome scan (genes in panel (d) were removed, such that only new target genes where represented here). Note that panels (b) to (e) are drawn to the same scale. **(f) **Portion of CNSs near to the genes in the different microarray clusters and embryonic development sets that is located within 200 base pairs (bp) of the transcription start site. **(g) **Portion of predicted CRMs near to the genes in the different microarray clusters and embryonic development sets that is located within 200 bp of the transcription start site. **(h) **Portion of CRMs predicted in a whole-genome scan for the transcriptional regulatory global model built for the different gene sets that is located within 200 bp of the transcription start site. The blue line in panels (f) to (h) indicates the portion of all CNSs (within 10 kilobases 5' of all human genes) that is less then 200 base pairs of the transcription start site. CI, Confidence Interval.

Because a gene's search space was defined as all CNSs within 10 kb 5' of the TSS, we first examined the distributions of CNS locations, because these represent the background distribution to which the CRM locations will be compared. A first important observation is that the CNSs are highly over-represented close to the TSS, as shown in Figure 5a,b. The type of gene set, namely adult tissue versus embryonic development, introduces a second CNS location bias (Figure 5c). Indeed, the adult tissue CNS set is enriched in sequences close to the TSS (<200 base pairs; *P *= 7.6 × 10^-16 ^by a Wilcoxon rank sum test), whereas the embryonic development CNS set is depleted in sequences close to the TSS and enriched in sequences further from the TSS (2,000 to 4,000 base pairs; *P *= 5.6 × 10^-7^). When evaluating each of the gene sets separately (Figure 5f), eight of the nine adult tissue CNS sets are enriched in sequences less than 200 base pairs from the TSS (in six cases, this was statistically significant by a χ^2 ^test), whereas all five embryonic development CNS sets are depleted in sequences less then 200 base pairs from the TSS (in three cases, this was statistically significant).

Next, we examine the location distribution of the CRMs that were identified by ModuleMiner. For adult tissue genes, CRMs are strongly over-represented close to the TSS (Figure 5d). Of these CRMs, 63% are within 200 base pairs of the TSS. In contrast, the CRMs that ModuleMiner identified near to the embryonic development genes are depleted close to the TSS and enriched further away (1,000 to 2,000 base pairs). These conclusions remain valid even when controlling for both biases mentioned above; comparing Figure 5d to Figure 5c (the predicted CRMs in Figure 5d can be considered a selection from the CNS sets in Figure 5c), the enrichment of predicted CRMs directing expression in adult tissues close to the TSS persisted (*P *= 2.6 × 10^-27^). (This was calculated as follows; the distances to the TSS of the predicted CRMs and all CNSs of the genes in the microarray clusters were ranked and the Wilcoxon rank sum test was applied.) For the CRMs directing expression in embryonic development, no statistically significant deviation from random selection from the embryonic development CNS sets could be identified (*P *= 0.18). When considering the gene sets separately, in eight microarray clusters expressed in adult tissues CRMs are enriched in sequences close to the TSS (Figure 5g; this was statistically significant when controlling for bias in six cases). In contrast, in four embryonic development gene sets, CRMs are depleted close to the TSS (markedly, for three of these sets, no CRMs were predicted within 200 base pairs of the TSS).

A similar difference in TSS distance distribution was also observed for the new target genes (Figure 5e). Here as well, the distances to the TSS of the CRMs predicted to direct expression in adult tissues were clearly nonrandomly distributed compared with all CNSs (*P *= 3.6 × 10^-74 ^by Wilcoxon rank sum test). For the CRMs predicted to direct expression in embryonic development, no statistically significant difference was observed (by Wilcoxon rank sum test). However, these sequences appear to be (slightly) depleted within 200 base pairs of the TSS (*P *= 1.5 × 10^-4 ^by a χ^2 ^test). Considering each of the gene sets separately (Figure 5h), in seven adult tissue microarray clusters, CRMs were significantly enriched within 200 base pairs of the TSSs, whereas for two embryonic development gene sets CRMs were significantly depleted close to the TSS. Although in six cases this effect was highly significant (*P *< 10^-9^), it was smaller than the effect within the clusters (compare Figures 5d and 5e).

In summary, the CRMs that ModuleMiner detected were nonrandomly positioned in the genome. CRMs predicted to direct expression in adult tissues were highly enriched very close to the TSS, whereas CRMs predicted to direct expression in embryonic development were depleted very close to the TSS.

## Discussion

Although the sequence of the human genome has been available for a considerable time now, our ability to chart the regions that control gene expression is still limited. The situation appears to improve as a function of smaller genome size. Indeed, in the *Drosophila *early segmentation network, CRMs can be predicted based on known examples [10,11]. In the yeast *Saccharomyces cerevisiae*, with a much smaller genome, it is possible to go one step further and predict the expression of genes based only on upstream sequences [36]. Here, we focus on the computational detection of CRMs in the human genome, and hence this work makes a contribution toward bridging this gap.

ModuleMiner detects CRMs by taking as input a set of co-expressed genes, under the assumption that a subset of these are co-regulated, and looking for a recurrent pattern of (computationally predicted) TFBSs. The advantages of this approach are that it does not require known examples and that it allows prediction of a probable function for the detected CRMs.

ModuleMiner is similar in scope to ModuleSearcher [20,29] and CREME [19]. It differs from these previous approaches in that ModuleMiner maximizes specificity for the given set of co-expressed genes by performing a whole-genome optimization. Indeed, ModuleMiner optimizes the combined rankings of the given gene set in a ranking of the complete genome. In addition, this approach allows comparison between TRMs with different parameters (for example, maximum CRM length, and number of PWMs in the TRM). Therefore, ModuleMiner can optimize over these parameters, and hence our approach effectively eliminates the need for parameters required by previous approaches.

Other algorithms have been developed that aim to detect similar CRMs in a set of co-expressed genes that (contrary to the approaches described above) do not use a library of PWMs [21,22,30,37]. Instead, and in addition to optimizing the combination of motifs, these algorithms optimize the motifs themselves. Hence, these methods attempt to solve a problem with considerably greater complexity, resulting in lower performance, as confirmed by our comparison on benchmark data. Given the extremely poor performance of motif detection methods in organisms other than yeast [38], we have opted to circumvent motif optimization by using experimentally determined PWMs. Note that this decision does not necessarily limit the search to known PWMs, because libraries of computationally predicted PWMs are also available (for example, the phylofacts PWM library [39]). In addition, we believe that with the emergence of the protein binding microarray technology [40], high quality PWMs will soon become available for a large fraction of the human transcription factor repertoire. Even though the currently available libraries of experimental PWMs exhibit high redundancy and may contain low quality PWMs, our new approach of clustering similar TRMs is able to group redundant PWMs, and our validations show that in many cases a combination of five experimental PWMs can capture enough information of a CRM to yield acceptable genome-wide specificity levels.

ModuleMiner outputs the predicted CRMs and a TRGM. This TRGM can be considered a bag of PWMs (selected from TRANSFAC and JASPAR), with a weight associated to each PWM. Therefore, this TRGM not only predicts the transcription factors that function in the process under study, but it also allows an assessment of the relative importance of each of these transcription factors.

TRGMs do not contain spatial relations between TFBSs (except for the total size of the CRMs and a Boolean parameter indicating whether different binding sites can overlap). Although certain spatial relations between transcription factors working in concert are known to exist (for example [41,42]), we did not find any reports indicating that this is the rule rather then the exception. Therefore, we reasoned that any such relationships should not be hard-coded into the TRGMs, but rather would become apparent by inspection of the predicted CRMs. Upon inspection of the predicted CRMs presented above, no such spatial relationships surfaced.

Our method for scoring a sequence using a TRM or TRGM (see Materials and methods, below) does not take homotypic clustering of TFBSs into account (like hidden Markov model based methods do [15,17,43]). However, this cooperative binding of one transcription factor can nevertheless be modeled in our framework by the construction of a TRM or TRGM that contains multiple instances of the same PWM. Therefore, if multiple instances of a specific transcription factor are important for the regulation of a set of co-regulated genes, then this is represented accordingly in the optimal model. For example, when applying ModuleMiner to the tightly co-expressed set of smooth muscle markers, the transcription factor SRF occurs two or three times in each of the TRMs in the resulting TRGM, suggesting an extensive cooperation between SRF binding sites for smooth muscle specific transcription regulation. In contrast, the SMAD4, SP1, and ATF3 PWMs occur exactly once in 97.5% of the TRMs (SMAD4 and SP1 occur twice in 1.5% and 1% of the TRMs, respectively).

ModuleMiner takes the genomic background sequence into account in two ways. First, a third order background model is used in the process of annotating putative TFBSs. Second, our optimization strategy selects the TRM (or TRGM) that optimally separates the given genes (sequences) from all other genes in the genome. Hence, our system corrects both for local sequence properties (by the third order background model) as for more global sequence properties (by selecting against combinations of TFBSs that occur independently of the given sequences).

We included all CNSs up to 10 kb 5' of the TSS in our pipeline. Although this choice is inherently arbitrary, it is motivated by the following arguments. First, sequences 3' of the TSS might harbor translational regulatory signals, which we do not wish to model here. Second, potential regulatory sequences far upstream can be difficult to assign to a target gene. Third, selecting 10 kb 5' of the TSS has proven to be valuable in our previous study [20], and others have made similar choices as well [44]. In a previous study, in which CRMs were predicted in an unbiased way across the complete human genome [8], it was shown that CRMs are highly depleted between 10 kb and 30 kb 5' of the TSS.

The validation framework that we use, combining genome-wide ranking with LOOCV, could also be useful in evaluating or comparing hypotheses regarding the working principles of transcription regulation, and in this regard can be considered similar in scope to CodeFinder [24]. In this work, two such tests are implicitly performed: CRMs driving a tissue-specific expression pattern are compared with CRMs driving an embryonic development expression pattern; and by comparing the three sets of putative TFBSs (for example, Figures 1, 3j, and 4b) the importance of binding site preservation is evaluated, as well as the impact of a correction for differences in TSSs between human and mouse.

Construction of a high-quality set of co-regulated genes involved in a certain process under study is not always straightforward. In this regard, robustness to noise in a set of putative co-expressed genes is highly desirable in an algorithm to detect similar CRMs. We found ModuleMiner to be highly robust to the quality of this input gene set. Indeed, in our experiments with smooth muscle marker genes we observed that ModuleMiner was able to pick up the correct signal even when only 10 out of 50 given genes are really co-regulated (Figure 2). These properties of ModuleMiner prompted us to apply the algorithm to gene sets obtained from clustering microarray data. In nine out of ten microarray clusters, ModuleMiner succeeded in finding similar CRMs in a subset of the genes. Perhaps unsurprisingly, a critical mass of co-regulated genes is required for ModuleMiner to detect similar CRMs. However, this minimum required number of co-regulated genes is sufficiently small so as not to preclude application of the algorithm. This is illustrated both by our results obtained on the smooth muscle genes (Figure 2) and by the successful CRM detection in two small heart development gene sets (Table 3).

Application of ModuleMiner to the smooth muscle marker genes resulted in CRMs with multiple binding sites for SRF, and with single binding sites for SMAD4, SP1, and ATF3. Both SRF and SP1 have been shown to play a role in regulating smooth muscle specific expression [27]. Furthermore, SMADs are effectors of the transforming growth factor-β signaling pathway, and have been shown to work in concert with SRF to control smooth muscle cell differentiation [45]. ModuleMiner identified transcription factors known to play a key role in other co-expressed gene sets as well. Examples are GATA factors, NFATs and HAND1 in heart development; HNF-1 and HNF-4 in liver-specific gene expression; PU.1 in lymphocyte specific gene expression; and myogenin, SRF, the thyroid hormone receptor, and MEF2 in heart-specific gene expression.

Imposing trans-factor conservation by motif preservation between human and mouse sequences of a CNS significantly improved the performance of ModuleMiner on the set of smooth muscle marker genes. A similar approach has also been shown to improve CRM detection performance in the *Drosophila *early segmentation gene network [10]. When we applied ModuleMiner to the microarray clusters and the embryonic development gene sets, in some cases this trans-factor conservation also increased performance (microarray clusters 6, 7 and 9, and the neural crest cell gene set), but in other cases it did not.

Correcting for possible differences in TSS in human and mouse by a three-step alignment procedure (see Materials and methods, below) resulted in increased performance for most of the microarray clusters, but not for the development gene sets. This marked difference may be related to the different locations of the detected CRMs in these two different systems.

We observed a significant difference in the locations of the CRMs ModuleMiner predicted to direct expression in adult tissues and the CRMs ModuleMiner predicted to direct expression in embryonic development. CRMs driving tissue-specific expression are highly over-represented within 200 base pairs of the TSS. In contrast, CRMs driving expression in embryonic development are more evenly distributed in the 10 kb sequences we considered, and appear to be under-represented within 200 base pairs of the TSS. These results suggest that transcription regulation of tissue-specific expression is mainly exerted by proximal promoters, whereas transcription regulation of expression during embryonic development appear mainly to be exerted by more distal enhancers.

ModuleMiner can be applied to three conceptually different tasks: prediction of transcription factors that play a role in regulating a set of co-regulated genes; prediction of regulatory regions; and predictions of new target genes of a TRGM. It is important to appreciate that the accuracy of predictions differs between those tasks. Although exact performance statistics can only be obtained through careful experimental testing of our predictions, which is outside the scope of the present study, the results we obtained in this work can be used to provide rough estimates of the predictive accuracy. When we applied ModuleMiner to the two well studied benchmark sets, we obtained HNF1, CEBP, HNF3, GATA1, PAX6 and HNF4 for the liver benchmark set; and MZF1, PPARγ, SRF, MEF2, the Epstein-Barr virus transcription factor R, MYF, and MYOD for the muscle benchmark set. Comparing this with the literature [4,46] and with the PWM libraries we use, we obtain a sensitivity of 70% (7/10 known PWMs are recovered), a specificity of 99.6% (630/633 [liver] and 619/621 [muscle] probably incorrect PWMs are rejected), and a positive predictive power of 62% (8/13 total predicted PWMs are correct). These values need to be regarded with some reservations when extrapolating to other cases, because both liver and muscle are well studied systems for which high-quality PWMs are available. Nevertheless, we can conclude that ModuleMiner is quite accurate in selecting PWMs/transcription factors that play a key role in regulating the genes under study.

Regarding detection of regulatory sequences, ModuleMiner was able to detect 16 out of 24 known muscle/liver enhancers, when a total of 24 predictions were made. This repesents a sensitivity of 67% and a positive predictive power of 67%, although we emphasize that this last value is an underestimate because some of our predictions may be yet unknown enhancers. Notwithstanding some reservations on extrapolating these data, we conclude that the predictive accuracy of ModuleMiner for detection of regulatory regions (CRMs) near to a set of co-regulated genes is quite high.

Regarding the predictive accuracy of ModuleMiner for the detection of new target genes given a TRGM, the results of our LOOCV procedure can provide some estimates. From the resulting ROC curves, one can see that for a sensitivity of 50%, the specificity is about 90%, and for a sensitivity of 80% the specificity is about 80%, although the differences between different gene sets can be large. However, typically only a few dozen new target genes can be tested, and thus specificity may not be high enough to select the right targets from the complete genome. In our previous study [23] we confirmed that the predictive accuracy of new target genes is quite low, although we showed it to be detectably present. We note that in that study we used our previous ModuleSearcher algorithm, which was shown here to have lower performance than ModuleMiner. In addition, ModuleMiner's use of network level conservation between human-mouse and rat-dog predictions of new target genes might increase performance. Finally, the results we obtained in the TSS distribution of the CRMs predicted near to the new target genes are consistent with these performance predictions; Figures 5e and 5h show a similar trend to that in Figures 5d and 5g but to a lesser extent, hence pointing to a substantial amount of noise, but also indicating that a signal can be picked up even in a whole-genome scan.

## Conclusion

We present ModuleMiner, the first algorithm to detect CRMs in the human genome that is based on whole-genome optimization. ModuleMiner is generally applicable and outperforms other similar approaches to detecting CRMs on benchmark data. In addition, ModuleMiner can detect similar CRMs in noisy sets of co-expressed genes, such as microarray clusters. We successfully applied the algorithm to sets of genes expressed in adult tissues and sets of genes expressed in embryonic development processes. We show that CRMs predicted to regulate genes expressed in adult tissues are highly over-represented within 200 base pairs of the TSS, whereas CRMs predicted to regulate genes involved in embryonic development processes are depleted within this region. These findings suggest that expression in adult tissues is mainly directed by proximal promoters, whereas expression in embryonic development is more often regulated by distal enhancers.

## Materials and methods

### Construction of three sets of candidate TFBSs

We constructed three sets of genome-wide candidate TFBSs in human-mouse CNSs. The first set contains all predicted binding sites in all CNSs. Sequences 10 kb 5' (+ 50 base pairs 3') of the TSS of all human genes and their mouse orthologs were obtained from Ensembl (version 36). When another gene was encountered, only the sequence up to that gene was included. CNSs were selected by LAGAN alignments [47]. Thresholds were set at 75% conservation over at least 100 base pairs. TFBS predictions were performed using MotifScanner [48], with the prior set at 0.2. Both TRANSFAC [49] (version 9.4) and JASPAR [39] were used as PWM libraries.

The second set aims to restrict the candidate binding sites by enforcing that the regulatory factors should be conserved. This is achieved by selecting only binding sites in each human region for transcription factors for which we also detect binding sites in the orthologous mouse region (preserved sites). We note that this constraint does not require the binding sites to be conserved or that they should align.

In the construction of the third set we aimed to correct for differences in human and mouse TSSs, and for possible annotation errors of TSSs. To this end, we extended the mouse sequences used in the alignments by 100 kb in both directions. Alignment errors were kept in check by applying a multi-step alignment procedure. The human 10 kb sequence was aligned to the 10 kb mouse sequence (alignment A), the mouse sequence extended by 10 kb in both directions (alignment B), and the mouse sequence extended by 100 kb in both directions (alignment C). If CNSs were predicted in alignment A, then we assumed that the correct orthologous region in the mouse is not off by more then 10 kb, and hence we used the CNSs from alignment A supplemented by all additional CNSs from alignment B. CNSs that were truncated in alignment A because they extended over the sequence borders were replaced by their counterpart from alignment B. If no CNSs were predicted in alignment A, then we reasoned that the correct orthologous region in the mouse might be off by more then 10 kb, and we used the CNSs from alignment C. Here also, for each CNS (in human) we selected only preserved binding sites.

The same procedure was used with the dog and rat sequences to create sets of candidate TFBSs corresponding to the three human-mouse sets. Because neither dog nor rat could serve as a reference species, we did not extend the sequences in the dog-rat candidate TFBS set that corresponds to human-mouse set 3.

### Transcriptional regulatory models

We model similar CRMs in a set of co-expressed genes by TRMs. These TRMs are parameterized as in the report by Aerts and coworkers [20]. A TRM is a combination of PWM instances (up to six), supplemented by three parameters: the maximum length of CRMs; a Boolean parameter stating whether different binding sites can overlap or not; and a Boolean parameter that indicates whether incomplete modules will be penalized. Given a TRM and a sequence, a score S_seq _can be calculated, as detailed in the report by Aerts and coworkers [20]. A TRM may contain multiple instances of one specific PWM. In the calculation of S_seq_, each PWM in the TRM is matched to at most one binding site; thus, if a PWM occurs twice, up to two binding sites for the corresponding transcription factor can be taken into account. We assign a score S_g _to a gene by taking the maximum of S_seq _for all CNSs of that gene. The S_g _scores for the given set of co-regulated genes are used to determine a 'fitness score' of a TRM. This fitness score of a TRM for a given set of co-expressed genes is determined by the positions of the co-expressed genes in a ranking of S_g _for all genes in the genome. We use order statistics to assign a probability to the combination of ranks of the given co-expressed genes (using the numerical approach detailed in the report by Aerts and coworkers [23]). Hence, the resulting *P *value represents how well that TRM models the given set of co-expressed genes, compared with all other genes in the genome. We use 1 minus that *P *value as the fitness score for the TRM.

### The ModuleMiner algorithm

ModuleMiner uses a genetic algorithm to find the TRM with the optimal fitness score. At the onset, a starting population of TRMs is obtained by running our ModuleSearcher algorithm [29] using many different combinations of parameters. This initial step is not absolutely required (one can start from a population of randomly generated CRMs), but it provides a speed advantage. These TRMs obtained by ModuleSearcher are assigned a fitness score, and the 200 best scoring TRMs are retained as starting population for the ModuleMiner genetic algorithm. During each 'generation' of the algorithm, 200 new individuals (TRMs) are generated (based on the TRM population at that time) and added to the population. This population of 400 TRM is then required to compete (by fitness score), and the 200 best scoring TRMs are retained. This procedure is repeated until the stop criterion is reached (at least 300 generations and at most 1,000 generations). Generation of new individuals (TRMs) is done using two 'parent' TRMs randomly selected from the population. Each of the TRM parameters (number of PWMs, length, overlap, and penalization) is determined by random selection from both parents, allowing a small probability of mutation (each parameter is set to a random value with a probability of 0.1). Subsequently, PWMs are selected at random from both parents. Here also, each PWM can be 'mutated' (replaced by a PWM randomly selected from TRANSFAC and JASPAR) with a probability of 0.1. As the stop criterion, we use homogeneity of the population: if more than 80% of the TRMs can be grouped into one TRGM (see below) and at least 300 generations have passed, then the algorithm is stopped. If this stop criterion is not reached, then the algorithm is stopped after 1,000 generations. The parameters of the ModuleMiner genetic algorithm (for example, population size, mutation probability, and so on) were selected by optimizing for speed. The convergence of the algorithm is highly insensitive to these parameters over a wide range, and sensitivity of speed to these parameter settings is also limited (data not shown).

### Transcriptional regulatory global models

Aiming to minimize the sensitivity of our models of similar CRMs to noise in TFBS predictions, we constructed composite models (TRGMs) from multiple high-scoring TRMs. To this end, similar TRMs are clustered, and the largest cluster is returned as resulting TRGM. TRMs were clustered when the CRMs they predict near to the high scoring genes (out of the given set of co-expressed genes) occur in the same CNS. As a cut-off for determining which genes are among the 'high scoring genes', we used the top 2.5% in a ranking of the complete genome.

Scoring a sequence with a TRGM is performed by scoring this sequence for each TRM within the TRGM, subsequently normalizing this score (maximum CNS score = 1), and finally adding the normalized TRM scores.

Because a TRGM is a collection of TRMs and TRMs each contain a collection of PWM instances, TRGMs are also collections of PWMs. In addition, a weight can be assigned to each PWM in the TRGM, quantifying the significance of the PWM for the process under study. This weight of a PWM is calculated as follows: for each TRM in the TRGM, the number of instances of that PWM is counted, and this number is averaged over all of the TRMs in the TRGM.

### Performance comparison on benchmark data

Four benchmark datasets containing annotated regulatory regions directing expression in a particular system were selected from PAZAR [28]. We selected all human genes (or human orthologs) from each of these 'boutiques'. The regulatory sequence search space was defined as all CNSs within 10 kb 5' of the TSS (as throughout our study). We used this search space for all algorithms, except CREME [19], for which only the online version was available that by default uses one CNS within 1.5 kb of the TSS. Because the other CRM detection algorithms had multiple parameters (absent in ModuleMiner), these parameters were set to default options. For the ModuleSearcher algorithm [29], we used the same parameters as in the cell cycle case study reported [20]. For CisModule [22] and EMCMODULE [30] we used the default parameter settings. We used Clover [31] as follows; for each PWM found to be over-represented, we constructed a TRM (with parameters; no overlap between binding sites, no penalization, and a maximum distance of 1,000 base pairs), and this way we constructed TRGMs containing enriched PWMs reported by Clover. We also generated 100 random TRMs (combinations of three to six PWMs with randomly generated parameters) and we used these to rank the genes of each benchmark set, as a proxy for a method unable to detect similar CRMs.

### Availability

ModuleMiner can be accessed at our website [26]. A stand-alone version is available upon request.

## Abbreviations

AUC, area under the ROC curve; CNS, conserved noncoding sequence; CRM, *cis*-regulatory module; GO, Gene Ontology; kb, kilobases; LOOCV, leave-one-out cross-validation; PWM, position weight matrix; ROC, receiver operator characteristic; TFBS, transcription factor binding site; TRGM, transcriptional regulatory global model; TRM, transcriptional regulatory model; TSS, transcription start site.

## Authors' contributions

PVL, SA, and PM conceived and designed the experiments. PVL performed the experiments. PVL and SA analyzed the data. PVL, SA, BT, BDM, and YM contributed reagents/materials/analysis tools. PVL wrote the paper. All authors read and approved the final manuscript.
